# Editorial: Facing the Upcoming of Multidrug-Resistant and Extensively Drug-Resistant Bacteria: Novel Antimicrobial Therapies (NATs)

**DOI:** 10.3389/fbioe.2021.636278

**Published:** 2021-02-23

**Authors:** Angel León-Buitimea, Jose Ruben Morones-Ramírez, Jason H. Yang, Rafael Peña-Miller

**Affiliations:** ^1^Facultad de Ciencias Químicas, Universidad Autónoma de Nuevo León, UANL, San Nicolás de los Garza, Mexico; ^2^Centro de Investigación en Biotecnología y Nanotecnología, Facultad de Ciencias Químicas, Universidad Autónoma de Nuevo León, Parque de Investigación e Innovación Tecnológica, Apodaca, Mexico; ^3^Center for Emerging and Re-Emerging Pathogens, Rutgers New Jersey Medical School, Newark, NJ, United States; ^4^Department of Microbiology, Biochemistry and Molecular Genetics, Rutgers New Jersey Medical School, Newark, NJ, United States; ^5^Center for Genomic Sciences, Universidad Nacional Autónoma de México, Cuernavaca, Mexico

**Keywords:** antibiotic resistance, multidrug-resistant bacteria, extensively drug-resistant bacteria, antimicrobial therapies, combinatorial treatments, metal nanoparticles, antimicrobial peptides

Antimicrobial resistance is one of the largest looming threats to global health (Friedman et al., 2016), increasing the morbidity and mortality associated with bacterial infections (Ventola, 2015). ESKAPE pathogens (*Enterococcus faecium, Staphylococcus aureus, Klebsiella pneumoniae, Acinetobacter baumannii, Pseudomonas aeruginosa*, and *Enterobacter* spp.) are responsible for the majority of nosocomial infections (Ma et al., 2020) and commonly “escape” the biocidal action of antimicrobial agents (Mulani et al., 2019). The frequent and increasing use of antibiotics in medical practice drives the emergence of multidrug-resistant (MDR) and extensively drug-resistant (XDR) pathogens (Magiorakos et al., 2012; Prestinaci et al., 2015). There is an urgent and critical need to design and engineer novel therapeutic alternatives for eradicating MDR and XDR bacteria through burgeoning technologies such as metal nanoparticles, genetic engineering, synthetic biology, peptide therapeutics, and combinatorial treatments. In this Research Topic, we assemble ten original articles highlighting recent discoveries around Novel Antimicrobial Therapies (NATs) against MDR and XDR bacteria.

Seven original research articles spanning diverse disciplines describe the development of NATs for clinically-relevant MDR pathogens. One study describes the one-pot synthesis of Ag-ZnO nanoparticles at low temperatures and demonstrated remarkable antimicrobial activity of these nanoparticles against methicillin-resistant *Staphylococcus aureus* (MRSA) (Naskar et al.). Another study achieved successful phytomedited synthesis of green TiO_2_NPs that proved to be effective for treating biofilm-based bacterial and fungal infections (Al-Shabib et al.).

Another research article assessed the therapeutic efficacy of antimicrobial combinations on carbapenemase-producing *Enterobacterales* (CPE). The authors showed how antimicrobial combinations synergized against most CPE expressing resistance genes. These antimicrobial combinations may facilitate the successful treatment of patients infected with CPE (Zhou et al.). Another original research article identified two potent combinations of antibiotics for clinical MRSA infection, both *in vitro* and *in vivo* (Yu et al.). A separate study found that the compound 2,4-Di-Tert-Butylphenol isolated from an endophytic fungus substantially reduced the secretion of virulence factors and biofilm and its associated factors controlled by quorum sensing in a dose-dependent manner in *Pseudomonas aeruginosa* (Mishra et al.).

Furthermore, a study tested the anti-virulence activity of potential uridine diphosphate glucose pyrophosphorylase (UDPG:PP) inhibitors and showed that these inhibitors are a potential drug candidates against *Streptococcus pneumoniae* infections (Cools et al.). New putative antimicrobial candidates were reported by Okella et al. They designed an antimicrobial peptide and performed target identification based on a putative antimicrobial peptide motif derived from fish. From all the peptide motifs generated in this work, the authors identified Pleurocidin (secreted by flatfish) as having strong antimicrobial potential.

Three review articles included in this special issue address the use of NATs to face MDR bacteria. A mini-review discusses combination treatments (particularly antimicrobial peptides and metal nanoparticles) as a pathway to develop antimicrobial therapeutics with broad-spectrum antibacterial action, bactericidal instead of bacteriostatic activity, and better efficacy against MDR bacteria (León-Buitimea et al.). Another review explored the possibility of designing antimicrobial nanoparticle-based devices to exploit the potential of antimicrobial nanoparticles to combat MDR pathogens (Gómez-Núñez et al.). Finally, a third review describes the mechanisms associated with drug resistance in pyogenic streptococci and discusses the advantages and limitations of innovative therapeutic strategies such as bacteriocins, bacteriophage, phage lysins, and metal nanoparticles (Alves-Barroco et al.).

In summary, this group of articles contributes to the search for new therapeutic strategies to combat antibacterial resistance. MDR and XDR infections are growing in incidence; the main challenges facing society are now to design, develop, and evaluate new therapeutic strategies that can spearhead the development of alternative therapies against clinically-relevant MDR pathogens.

## Author Contributions

All authors listed have made a substantial, direct and intellectual contribution to the work, and approved it for publication.

## Conflict of Interest

The authors declare that the research was conducted in the absence of any commercial or financial relationships that could be construed as a potential conflict of interest.

## References

[B1] Friedman N. D. Temkin E. Carmeli Y. (2016). The negative impact of antibiotic resistance. Clin. Microbiol. Infect. 22, 416–422. 10.1016/j.cmi.2015.12.002 26706614

[B2] Ma Y. X. Wang C. Y. Li Y. Y. Li J. Wan Q. Q. Chen J. H.. (2020). Considerations and caveats in combating ESKAPE pathogens against nosocomial infections. Adv. Sci. 7:1901872. 10.1002/advs.201901872 31921562 PMC6947519

[B3] Magiorakos A. P. Srinivasan A. Carey R. B. Carmeli Y. Falagas M. E. Giske C. G.. (2012). Multidrug-resistant, extensively drug-resistant and pandrug-resistant bacteria: An international expert proposal for interim standard definitions for acquired resistance. Clin. Microbiol. Infect. 18, 268–281. 10.1111/j.1469-0691.2011.03570.x 21793988

[B4] Mulani M. S. Kamble E. E. Kumkar S. N. Tawre M. S. Pardesi K. R. (2019). Emerging strategies to combat ESKAPE pathogens in the era of antimicrobial resistance: a review. Front. Microbiol. 10:539. 10.3389/fmicb.2019.00539 30988669 PMC6452778

[B5] Prestinaci F. Pezzotti P. Pantosti A. (2015). Antimicrobial resistance: a global multifaceted phenomenon. Pathog. Glob. Health 109, 309–318. 10.1179/2047773215Y.0000000030 26343252 PMC4768623

[B6] Ventola C. L. (2015). The antibiotic resistance crisis: part 1: causes and threats. P T 40, 277–283. 25859123 PMC4378521

